# State-Transition Diagrams for Biologists

**DOI:** 10.1371/journal.pone.0041165

**Published:** 2012-07-23

**Authors:** Hugues Bersini, David Klatzmann, Adrien Six, Véronique Thomas-Vaslin

**Affiliations:** 1 Universite Libre de Bruxelles, Brussels, Belgium; 2 Université Pierre et Marie CURIE University Paris, Integrative Immunology: Differentiation, Diversity, Dynamics, Paris, France; National Research Council of Italy (CNR), Italy

## Abstract

It is clearly in the tradition of biologists to conceptualize the dynamical evolution of biological systems in terms of state-transitions of biological objects. This paper is mainly concerned with (but obviously not limited too) the immunological branch of biology and shows how the adoption of UML (Unified Modeling Language) state-transition diagrams can ease the modeling, the understanding, the coding, the manipulation or the documentation of population-based immune software model generally defined as a set of ordinary differential equations (ODE), describing the evolution in time of populations of various biological objects. Moreover, that same UML adoption naturally entails a far from negligible representational economy since one graphical item of the diagram might have to be repeated in various places of the mathematical model. First, the main graphical elements of the UML state-transition diagram and how they can be mapped onto a corresponding ODE mathematical model are presented. Then, two already published immune models of thymocyte behavior and time evolution in the thymus, the first one originally conceived as an ODE population-based model whereas the second one as an agent-based one, are refactored and expressed in a state-transition form so as to make them much easier to understand and their respective code easier to access, to modify and run. As an illustrative proof, for any immunologist, it should be possible to understand faithfully enough what the two software models are supposed to reproduce and how they execute with no need to plunge into the Java or Fortran lines.

## Introduction

**Figure 1:**
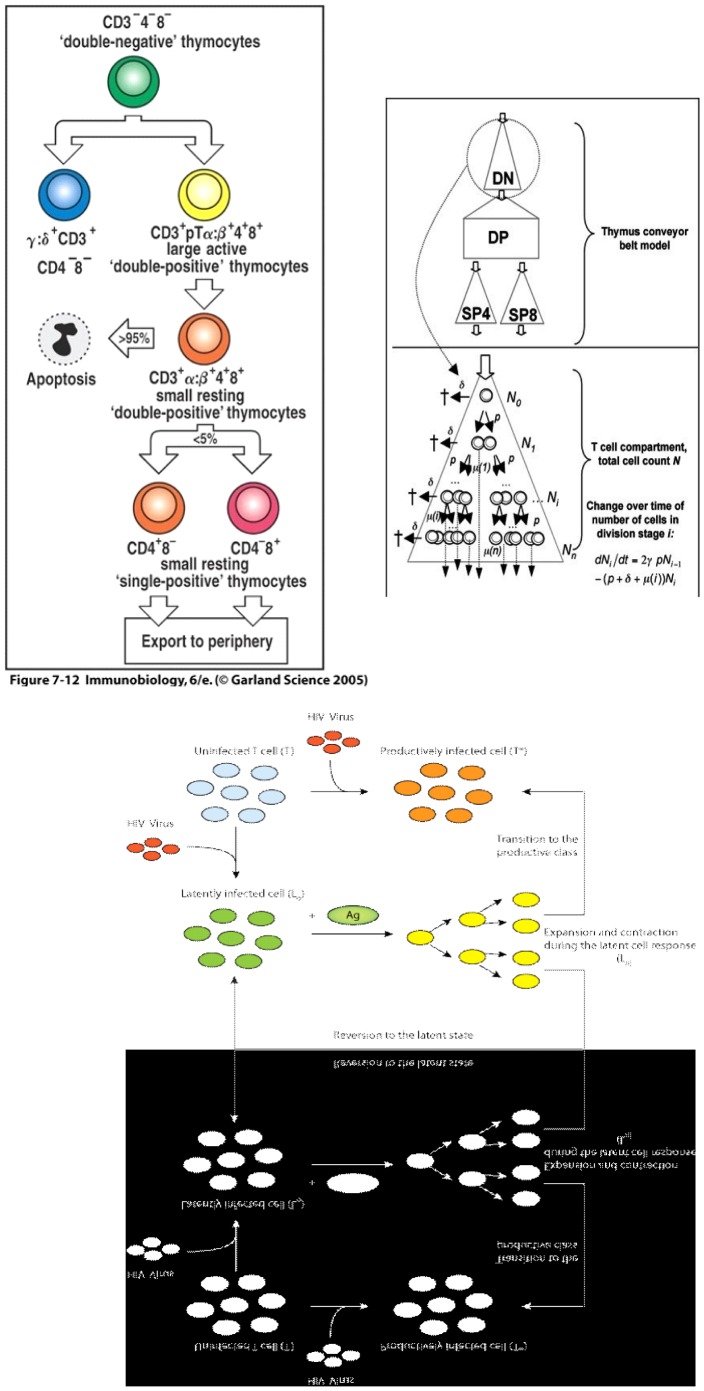
Three graphical illustrations of immunological knowledge which are very similar in spirit to state-transition diagrams: extract from Janeway’s classical immunology textbook [**22**] illustrating the successive thymocyte differentiation stages - extract from a paper by Veronique Thomas-Vaslin et al. [15] illustrating the conveyor belt model of thymocyte differentiation to be discussed later - extract from a paper by Rong and Perelson [23] illustrating the successive infection stages by the HIV virus.

It is clearly in the tradition of biologists to conceptualize the dynamical evolution of biological systems in terms of state-transitions of biological objects, as illustrated in Fig. 1. For example, at levels of gene molecules or cells, an object in an “inactive” stage, if receiving enough stimulation by external signals, will switch into “active” stage. After a given period of time and reaching a specific environment, a cell will “differentiate” and thus switch from one cell phenotype to another. In the figure as in the rest of the paper, and although biology as a whole is indifferently targeted, we mainly concentrate on its immunological branch since the ideas presented and defended here have been essentially discussed and experimented with immunological partners. As a matter of fact, it is definitely a stream of biology that has a long tradition of software and mathematical modeling and could consequently be more receptive to the proposals of this paper. Among examples of state transition are: during its early stay in the thymus, a pro T-cell is subject to a succession of differentiation steps, such as DN (Double Negative) then DP (Double Positive) to finally export mature T-cells (Fig.1a,b) - a viral encounter that turns an healthy target cell into an infected one - a T lymphocyte that, by encountering this same infected cell, switches from a naive state to an effector one to finally a memory one.

The state-transition diagram originally proposed by David Harel [1], and developed to specify safety-critical control software in the avionics industry, has become one of the many UML (Unified Modeling Language [2], [3]) standardized diagrams and, definitely, one of the most popular and useful ones in the professional software world. David Harel has become a vocal and active proponent of using state-transitions diagrams (also called statecharts through the paper) for biological modeling (see e.g. [4]–[6]).

Although the majority of researchers interested in biological software modeling increasingly agree that the most natural way to program their models is to adopt Object-Oriented (OO) practices, UML diagrams are still largely absent from their publications. However, in the last 15 years, the use of UML has risen constantly, to the point where it has become the de-facto standard for graphical visualization of software development. UML and its 13 diagrams has many universally accepted virtues. Most importantly, it provides a level of abstraction higher than that offered by OO programming languages (Java, C++, Python,.Net) that encourages researchers to spend more time on modeling rather than on programming.

Many indicators are pointing to UML as the natural next step up the abstraction ladder in the evolution of software development. Firstly, there is the ongoing multiplication of platforms that can reverse-engineer code from UML diagrams. Examples at different levels of sophistication include Rational Rose, Together, Rhapsody, Omondo and Altova. All major development CASE tools, in whatever popular programming language, such as Visual Studio or Eclipse, offer facilities to synchronize the code production and the drawing of associated UML diagrams. It is still an ongoing topic of debate just how far this synchronization should go. James Rumbaugh, one of the three original UML authors, criticizes the current evolution of UML while claiming in [7]: “I think a lot of the recent work on UML has been misguided. It never was meant to be a programming language. Use it to get the strategy right and write the final program in a suitable programming language”.

Today, in the software community, we can draw an imaginary axis along which to settle the diverging positions regarding UML. At one end of this axis are programmers sketching some very simple and informal diagrams on a whiteboard, to gain perspective on their own code, to take a little detachment from the code writing, to communicate what the code does, to document their production or to serve as a basis for discussion about coding strategies. Users at this end of the axis are occasional and light UML users, spending most of their time producing lines of code and who will only use a maximum of 20% of UML’s graphic symbols. The “agile programming” community can be counted among this class of users. On the other end, there are more and more developers that regard UML as the next generation of programming language, and actually generate most of their code automatically through UML diagrams and code generation tools.

Nevertheless, UML is here to stay. There is one and only one UML, it is the only standardized modeling graphical language and as such benefits from the support of all the big computer and software companies that have integrated UML in their product. It transcends any programming language and any computer platform and encourages to spend more time on modeling than on writing code. Its usage is constantly increasing among developers, so that we can only regret the minor diffusion of this graphical language among researchers producing biological software models and hope that this paper will improve the situation in the years to come.

Although there is a more than 20 years old tradition of immune software models, very few of them have been the object of further running and exploitation once their authors published the paper describing it and even made the code easily available. For most of the researchers, the amount of new knowledge acquired by painfully understanding and running the code does not deserve such cognitive expenses. If forced to run it, they will use and run it just as a black box. Again the adoption of UML and more particularly the state-transition diagram should improve the current situation. As a first but key role, these diagrams could be construed as some form of entrance door to the final code, also made available, whatever proximity or homeomorphism exists between these models and this code.

The abstract of a recent Nature paper [8] urgently claims:

“Scientific communication relies on evidence that cannot be entirely included in publications, but the rise of computational science has added a new layer of inaccessibility. Although it is now accepted that data should be made available on request, the current regulations regarding the availability of software are inconsistent. We argue that, with some exceptions, anything less than the release of source programs is intolerable for results that depend on computation. The vagaries of hardware, software and natural language will always ensure that exact reproducibility remains uncertain, but withholding code increases the chances that efforts to reproduce results will fail.”

Although any mention of UML is absent, we believe that this reasonable claim is pointing towards the necessary complementary provision of UML diagram descriptions of how the released codes are organized and how they are supposed to be executed. Entering into a code through the UML diagrams largely decreases the burden of adopting and running someone else software production.

For most of the programmers and UML adopters, state-transition diagrams apply to a single object. In principle, this type of diagram aims at following the state-transitions of one complicated class of agent over its lifetime. It indicates all possible states an object can be found in and all possible transitions (which can result from an external event or based on some internal conditions beyond threshold) between these states. In contrast to the class and sequence UML diagrams, no clear and unanimous proposal has emerged on ways to automatically map the state diagram into source code. If “class” and “message” have their obvious semantic counterpart in any OO programming language, this is no longer the case for “state” and “transition”. Some software suites, like IBM Rational Rhapsody (whose use is largely advocated by Harel [4]–[6]), use custom solutions to generate code from the state-transition diagram. Alternatively, the state design pattern [3], [9] offers one possible way to automatically associate the preceding state-transition diagram with a possible corresponding class diagram mapping each state onto a class responsible for the behavior of the agent while in that state. Each state will also be responsible for taking care of the possible transitions occurring from that particular state.

In software models of biological systems, traditionally a state-transition diagram is more likely to be translated into an agent-based model in which one single biological object and its successive transitions are followed in time. Although we will equally refer to this very natural and classical translation in this paper, the main and most original part of its content will be dedicated to a complementary interpretation of this diagram in terms of population and Ordinary Differential Equations (ODE). In such case, we rather are in presence of various populations of similar objects whose transitions are followed in time. Here state transition must rather be interpreted in a stochastic way and only a subpart of the objects in a given state transits to the following state. A random sampling of the population in the current state disappears from that same state to move to the next one. In the paper, we will refer to these two alternative interpretations as 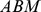 (Agent-Based Modeling) and 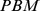 (Population-Based Modeling).

Due to the huge number of immune cells present in an organism, and following the tradition of chemists who rather privilege the use of population-based kinetics to ABM description of how molecular objects move, meet and react in time, a PBM approach has been favored in this paper so as to be more faithful to quantities found in reality and to be much more computationally effective. Noteworthy, though, the state-transition diagram per se is not really affected by this choice and would look very much the same whatever choice is being adopted: ABM or PBM. Nevertheless, most of the paper is dedicated to a formal translation of UML state-transition diagrams into a PBM grounded on ODE description (referred in the rest of the paper as 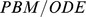).

There is a long ongoing debate in many scientific communities regarding the use of agent-based approaches versus population-based ones. Recent works critically analyze and compare the many ways to simulate immunology: ODE (deterministic and stochastic), cellular automata, ABM and even some forms of hybrids among them, before making their own interesting proposals [10]–[12]. For instance, in an attempt similar in spirit to the two models to be described later in the paper [10], the authors expose a conceptual workflow model of the immune system response they want to simulate in terms of logical flow, interactions among the immune actors and state-change affecting them. However, by non adopting a standardized graphical modeling language, it is hard to formally or automatically map this qualitative model into the sophisticated ABM model that they describe later in that same paper. Even aware of this conceptual model, even fully grasping it, any theoretical immunologist interested in better understanding the simulation results or even replicating them will not avoid the laborious reading and “decoding” of the proposed simulation code. The gap between the workflow and the final code remains too deep.

Although a possible automatized translation of UML state-transition diagram into either an ABM software or a PBM/ODE one is under intensive development [13], [14], as an alternative to Harel’s exclusive usage of IBM proprietary Rhapsody, this paper (aware of the existence of the axis separating the UML users mentioned early), deliberately avoids to adopt an authoritarian and definitive position about the right use of state-transition diagrams on the road to the final software. Rather it just argues for its increasing use and notices the parallel easy to draw between these diagrams and ABM or PBM production. Briefly, and although adopting immunology as a kind of discipline guinea pig, this paper might be justified at the crossroad of three current increasing trends in most biological disciplines: 1) A large part of the biological knowledge is captured in terms of state-transitions - 2) More and more ODE/PBM software models are being produced but very rarely elaborated and re-used beyond the authors original production - 3) The UML state-transition diagram has been standardized and is increasingly adopted by software developers.

The organization of the paper is as follows. The following sections will didactically describe the main graphical elements of the state-transition diagram and how they can be mapped onto a PBM/ODE corresponding mathematical model. Although this preliminary pedagogical presentation will not concern any precise biological reality, the last section will concentrate on two already published immune models of thymocyte behavior and time evolution in the thymus. The first one was originally conceived as a PBM/ODE whereas the other as an ABM. Although readers are referred to the original papers in order to fully grasp the content and the running of the two codes, we will sketch how both could be refactored and expressed in a state-transition form so as to make them much easier to understand and their respective code easier to access, to modify and run. For any immunologist, it should be possible to understand faithfully enough what the two software models are supposed to reproduce and how they execute with no need to plunge into the Java or Fortran lines.

## Methods

### Elementary Transitions

**Figure 2:**
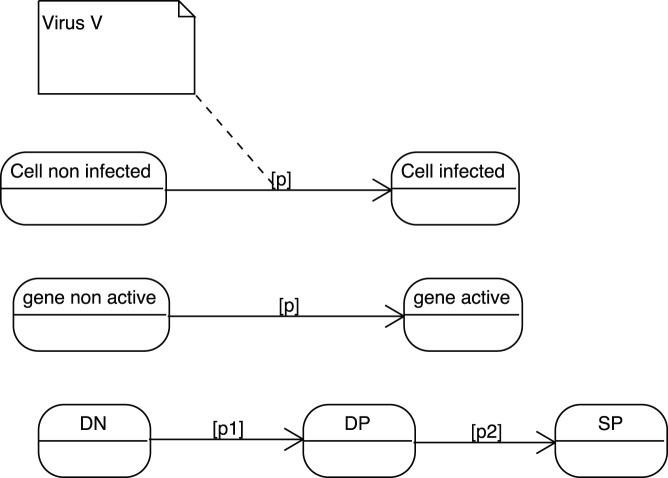
Three classical elementary biological state transitions. A cell is being infected by a virus, an inactive gene becomes active and a thymocyte switches between differentiation stages.

Suppose the three classical and independent biological elementary transitions illustrated in Fig.2 (all figures have been done by exploiting various UML software. Although some slight graphical differences might appear here and there, such as the exact shape of the “state” rounded rectangles, they are all realized according to the UML standard.). A cell turns out to be infected by the presence of a given virus, with a probability p - an inactive gene becomes active with a probability p (this could occur in presence of a given signal or a protein that we leave out of the explanatory scheme for simplicity) - a T-cell differentiates from type DN to DP with probability p1 and from DP to SP with probability p2. An ABM model version of the first transition could go that way. The given cell could move randomly around a coded 2-D or 3-D virtual environment and, by encountering a virus moving in that same environment, adopts the infected state with probability p. Similarly, a gene could, at each simulation time step, turns active with probability p. On the other hand, an ODE version of the first state-transition diagram would rather look like:
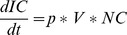
(1)

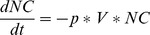
(2)with 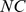 the number or the concentration of the non infected cells, 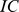 of the infected cells and 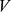 of the virus. This would describe a very typical model of viral infection in which the rate of infection is proportional to the number of 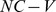 pairings i.e. a classical mass-action approach.

The ODE version of the third state-transition diagram could simply look like:
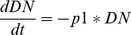
(3)

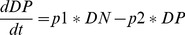
(4)

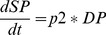
(5)One clear advantage of the graphics as compared to the mathematical translation resides in the economical treatment of the transitions that appear twice in the ODE, negatively on one side and positively on the other side. It ought to be much easier and convenient for a biologist to test the values of these probability parameters by directly accessing and modifying them on the graphics instead of manipulating them in the mathematical writings or in the program. This type of economical representation will occur many times in the rest of the paper and turns out to be one key argument in favor of this quite compressed graphical expression of biological transitions (with no loss of information to be deplored). The absence of any time sequencing is another obvious advantage of this ODE translation. In a PBM version of the state transition diagrams, all transitions occur simultaneously. Here the transitions are deterministically expressed through the presence of time rates. Nevertheless a stochastic translation is as well possible in which case the transition becomes stochastic with a random sampling of the population in a first state to switch to the second state. It is left to the biologist modeler to decide which mathematical time evolution is being favored, deterministic or stochastic, with no impact on the diagram.

**Figure 3:**
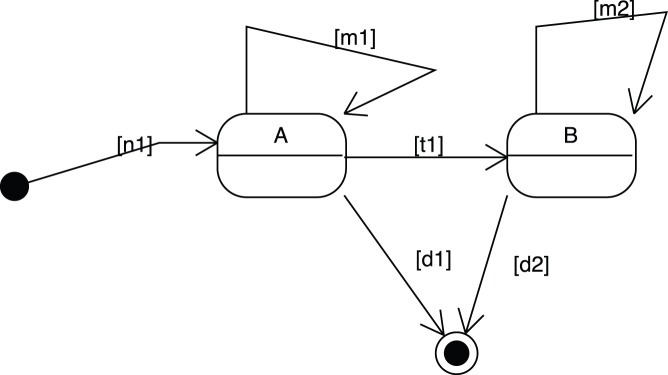
A more faithful and complete use of state-transition diagrams in the presence of origin and death states with possibility of proliferation and transit from stage A to B.

A more faithful and complete use of state-transition diagrams should rather produce graphics of the kind represented in Fig.3.

Additional graphical elements are the 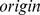 (with value 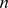) and 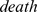 (with value 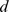) states and some internal transitions that, here, could just represent cell mitosis (with values 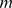). An ODE translation of this state transition diagram could look like:
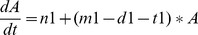
(6)

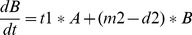
(7)In this mathematical mapping, for sake of simplicity, only linear expressions have been considered but nothing prevents a transition to adopt a more sophisticated mathematical non-linear form. For instance, the transition from A to B could be subject to a different mathematical treatment expressed by a function 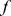 (that could appear in a way or another on the state-transition diagram or taken to be implicit and hidden in a box of the CASE tool) so as to have:
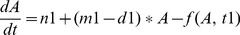
(8)

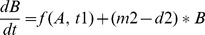
(9)


We leave as an open issue so far how detailed should the state-transition diagram be on the way to the final ODE (for instance, the “f” function discussed above or the type of numerical integration, the integrative time step…).

**Figure 4:**
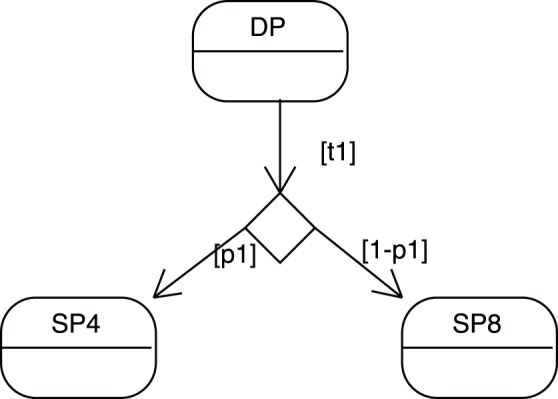
A state-transition diagram illustrating a conditional transition. Here a DP cell can alternatively differentiate into either a SP4 or SP8 cell with respective probability 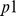 and 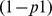.

A transition can even be conditional such as in Fig.4, in the case a DP cell can alternatively differentiate to either a SP4 or a SP8 cell with respective probability 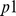 and 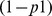.
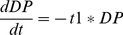
(10)

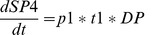
(11)

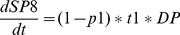
(12)


### Macrostate and Macrotransitions

One nice graphical addition of state-transition diagrams, also giving rise to a welcome economical representation, is the presence of compound states and macrotransitions that can be factorized over all micro states composing the macro one. For example, suppose that a model follows the course of one student in computer science during his university years. The student can succeed or fail (so only two states “in” and “out” would characterize this preliminary attempt) and a probability of failing be considered in the model. Now suppose you realize that this probability is too imprecise to match the real situation, that the model is far too approximate and that this failure rate really depends on the academic year (5 successive years for instance), the probability being much higher in the first year than in the fifth. In such a case, the “in” state should be further decomposed into 5 successive years/states, with a macro failure rate now depending on the academic year and 4 additional probabilities characterizing the successive transitions between these academic years.

**Figure 5:**
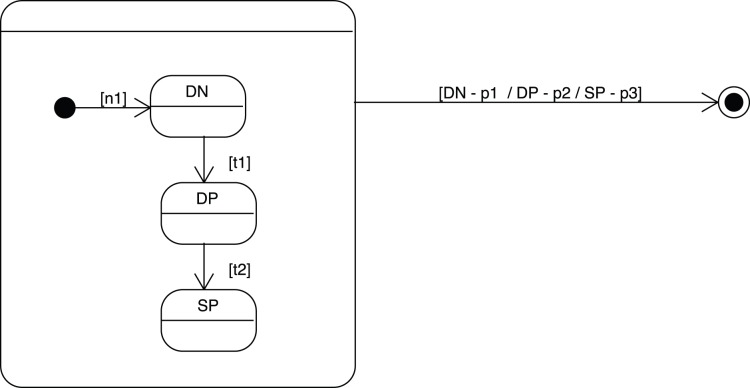
Example of compound state-transition diagram containing three single states and internal transitions. A common transition leaving the compound state is shared by all single states although presenting different transition rates.

As illustrated in Fig.5, this compound state provides a graphical representation where similar functionality, e.g. transitions, shared by all single states or sub-states can indeed be factorized so as to minimize diagram clutter. This hierarchical organization additionally allows the modeler to “zoom” into specific levels of model detail and adapt his model to the reality by adjusting his observation lenses. A simple state may be expanded into a compound state as more relevant detail about the system becomes available (for instance new transitions among the micro states).

The mathematical translation of Fig.5 could go as follows:
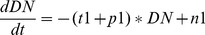
(13)

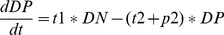
(14)

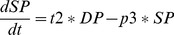
(15)


Here the death rate 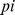 (and more generally the macrotransition rate) might depend on the respective single states but nevertheless one unique subsequent state remains common to all single states included in the macro one.

### Parallel State Transitions

**Figure 6:**
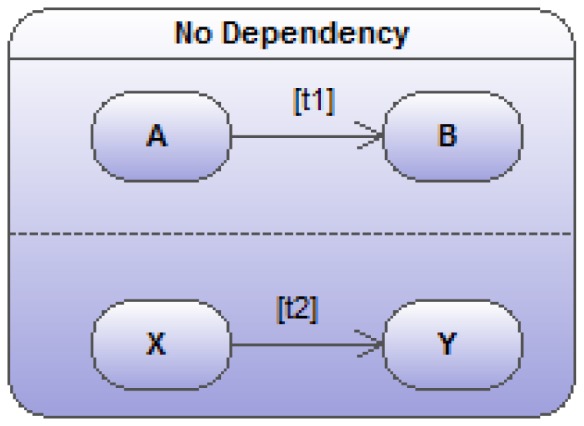
Two fully independent sub state-transition diagrams (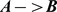 and 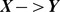) executing in parallel.

As illustrated in Fig.6, another optional possibility of UML state transition diagrams that, more than anything else before, once again allows a huge representational and graphical economy, is the presence of parallel transitions. The original idea underlying such possibility is the existence of simultaneous while distinct activity or state flows of a singular object. For instance, an university student can simultaneously move from academic year to academic year while, during that same period, integrate a sport-specific institution or modify his civic status by getting married. All these three life flows occur simultaneously like on three parallel tracks (independent but simultaneous in time).

In a more biological context, a cell can differentiate while moving between different body compartments. The combinatorial nature of these parallel transitions is the main reason behind this parsimonious representation in several parallel tracks. In the case of our student, many transitions should be taken into account (for instance suppose 5 successive academic years * 2 (for integrating or not the sport institution) * 2 (for the change in civic status)). Among possible transitions, our student in the 3rd year of his studies could get married, being married he could move to the fourth year, being in the fourth year he could integrate or leave a sport academy…. Over 20 combined states have to be considered and as many possible transitions to connect them.

### Basic Independent Transitions

Suppose, as illustrated in Fig.6, the two transition sub-diagrams (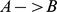 and 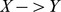) to be fully independent. An obvious mathematical translation of the 2×2 transitions among the 2×2 combined states could go as follows:
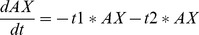
(16)

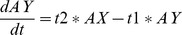
(17)

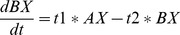
(18)

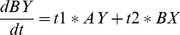
(19)This parallel state-transition diagram remains the simplest representation of something that would be graphically and organizationally unwieldy due to its intrinsic combinatorial nature. Moreover, the value of this graphical representation of parallel transitions is that they provide a manner to specify just the deviation from independence, while diagrammatically representing the sub-systems as if they were truly independent. Let’s envisage some of these deviations from this total independence rather unrealistic in many biological contexts.

**Figure 7:**
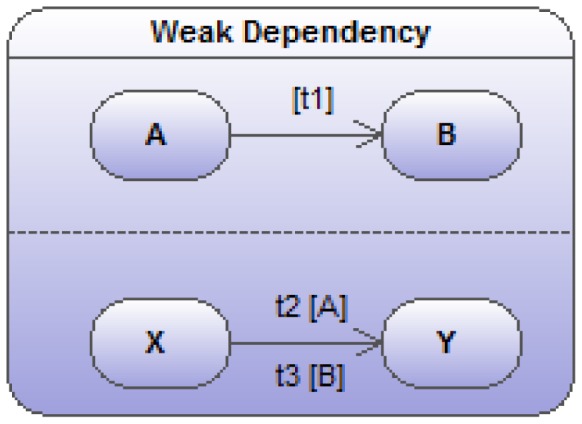
Two sub state-transition diagrams (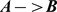 and 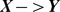) showing a weak dependency. The transition rates in the lower track depend on the state in the upper track.

**Figure 8:**
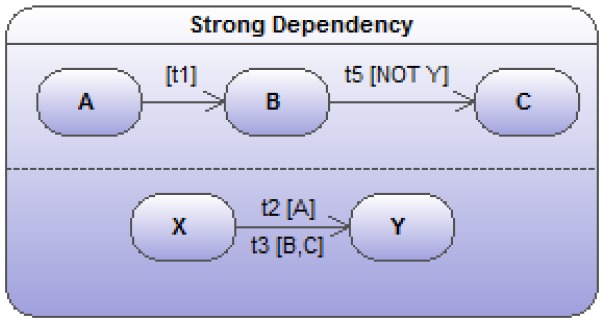
Two sub state-transition diagrams (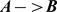 and 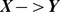) showing a strong dependency. When the object is in state 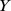 in the lower track, the transition 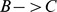 becomes impossible. This is indicated by conditioning this transition with *[NOT Y]*.

### Weak Dependencies

In the updated version of Fig.6 depicted in Fig.7 (and much more respectful of biology in general), the two transitions are not fully independent. The value of the transition rate in one track turns out to be dependent on the state in which the object is being found in the other track. Here, for instance, the 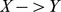 transition rate depends on the current state of the object of interest in the parallel 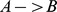 sub state-transition diagram. The new mathematical translation is as follows:
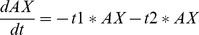
(20)

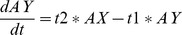
(21)

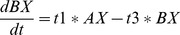
(22)

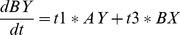
(23)


### Strong Dependencies

A stronger form of dependency (also quite common in biology) occurs if a transition in one of the parallel sub-diagrams is made impossible by the object being in a given state in another parallel sub-diagram such as indicated in Fig.8. In the figure, the 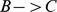 transition is rendered impossible by the object finding itself in state 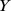. The mathematical translation needs to be updated as follows:
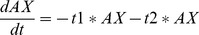
(24)

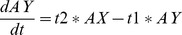
(25)

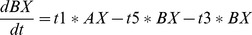
(26)

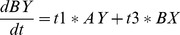
(27)

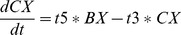
(28)

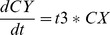
(29)


**Figure 9:**
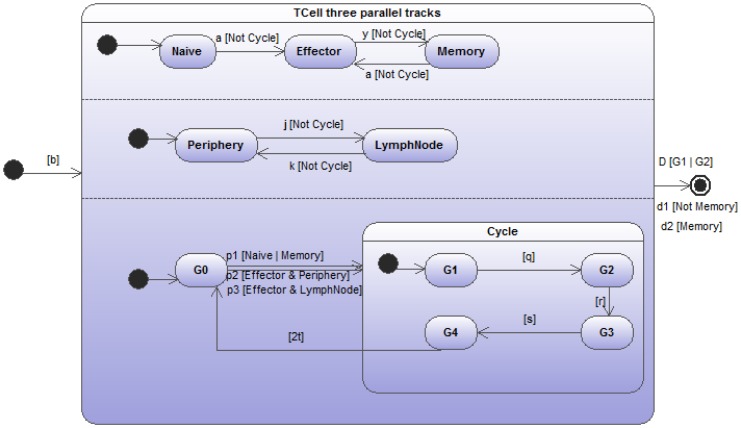
As a more realistic example of weak and strong dependencies, the figure illustrates three parallel sub-state-transition diagrams of a very classical immunological situation with lymphocytes *differentiating*, *migrating* and *dividing* (in 3 successive stages). When entering its dividing cycle, all other transitions turn out to be impossible. This is indicated by the presence of *[Not cycle]* with 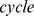 labeling the compound state of dividing cell. Some of the transition rates “weakly” depend on the states where the lymphocyte is currently found in the other parallel tracks. 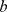 indicates the birth rate to naive cells.

As a clear illustration of this type of strong dependency, the Fig.9 presents a much more realistic immunological situation describing three respective and simultaneous dynamical evolutions of lymphocytes: *differentiation, migration and proliferation*. Lymphocytes, following an initial period of “naivety”, have their receptor bind to a ligand. This induces the cells to become “effector”, performing some function (of no relevance here). After a given period, effector functionality wears off and this binding process can repeat indefinitely, but the cells are now upgraded to “memory”. Once in their memory state, cells can become effector again (this transition might even be faster than in the initial naive case). Such models are typical in theoretical immunology with parameters 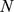, 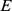, and 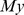 representing naive, effector and memory cell populations, respectively. 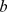, 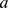 and 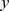, 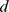 are rates of birth into naive cell, differentiation into effector, memory state and death (characterizing the first parallel track), respectively. Moreover, for the 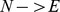 and 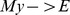 differentiation rate 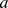, we assume a non-linear function that quantifies the competition for binding to ligand 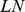, the details of which have no immediate bearing on our discussion.

Additionally, lymphocytes spend their life between peripheral tissues (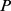) and the lymph nodes (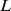), where antigenic debris is drained and collected to be exposed to lymphocytes. So the second parallel track of the whole diagram just accounts for this transfer between the lymph nodes and the peripheral tissues (with rates 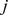 and 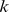).

Finally (i.e. the third track) cells cycle in a repetitive succession of 3 stages (classically labeled 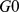 - the quiescent stage, 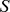 and 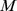) after which they give rise to a clone (i.e. the 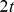 characterizing the transition between the final stage of the cycle 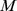, then the duplication and the start of a new cycle 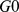). What is fundamental here, as a further illustration of a strong dependence among the parallel evolutions but quite classical in biology, is that once a cell enters its dividing cycling process, all other parallel state transitions are blocked. For instance, a T-cell can move from one site to another only when being in the 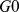 state, but this displacement becomes impossible once entering its 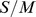 cycling process.

Translating this parallel state-transition diagram into an ODE mathematical form, like done in the following, reveals the enormous complexity of the underlying dynamics and the incredible simplification allowed by the recourse to state-transition diagrams. Normally, 18 equations are necessary as an outcome of this translation. For sake’s of simplicity, we limit ourselves to show only 4 of these 18 equations, the others being very easy to deduce. The different cell populations to be simultaneously followed in time are designated by combining the different states extracted from the 3 parallel tracks (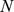 and 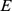 and 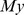; 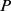 and 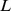; 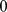 and 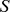 and 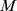 for the cycle stages). As can be easily deduced from observing the figure, some of the transition rates are clearly dependent upon which state in the two other tracks the cells find themselves at.
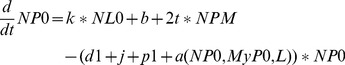
(30)

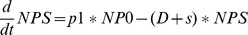
(31)

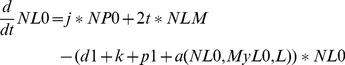
(32)

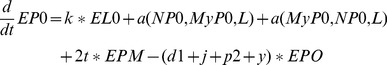
(33)


### Coupled Transitions

**Figure 10:**
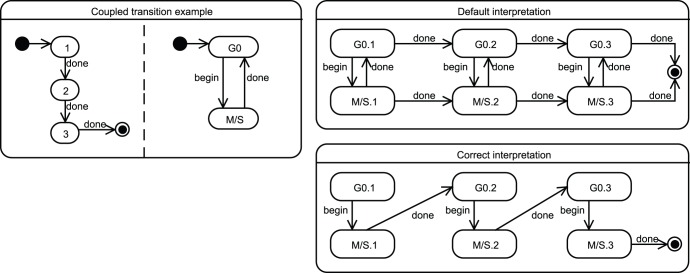
An illustration of a coupled transition (*existing*) in parallel tracks, representing a simple cell division and differentiation (left). As cells transit from the 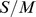 cycling stage back to state 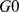, they should *also* move through the successive differentiated types - 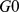, 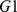 and 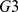 are three successive generations of cells. The default interpretation goes as the top-right figure but should rather be transformed into the bottom-right due to the shared labeling 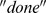 of the coupled transitions.

Unfortunately, this transition reduction is not totally sufficient to properly express important statechart semantics. Other very common biological dependencies between parallel evolutionary tracks are transitions that have to be synchronized in time. For instance, when moving from the third academic year to the fourth one, a European student switches from the bachelor to the master status and these two transitions should be coupled so as to simultaneously happen. Figure 10 illustrates the problem with a parallel statechart representing, one the one hand, a simple cell division (a cycle restricted to two stages) and, on the other hand, a 3-type differentiation process. Clearly, these two tracks are not independent: it is when cells transit from the 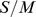-phase back to state 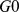 that they should simultaneously move between two differentiation stages. The two right halves of Fig. 10 illustrate the differences in the directed graph that might be indifferently generated and the correct one truly intended by the statechart semantics. The easiest way to mark these coupled transitions is by giving to transitions that need to simultaneously occur a same label (as illustrated in the figure with the label 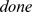).

## Results

In the following, we will sketch how existing immune models of a very similar immunological situation (thymocyte differentiation in the thymus) should gain in readability and accessibility as an outcome of the graphical representation under the form of state-transition diagrams. This exercise will be performed for two existing and published models: one that gave rise to a PBM/ODE software and the other an ABM software. It would be much too long and redundant to describe in details the behavior of these two models. The main reason for this last section is to advocate a possible understanding and manipulation of them both with no need to get into the final code.

### State-transition Description of a Thymocyte Differentiation PBM Model

**Figure 11:**
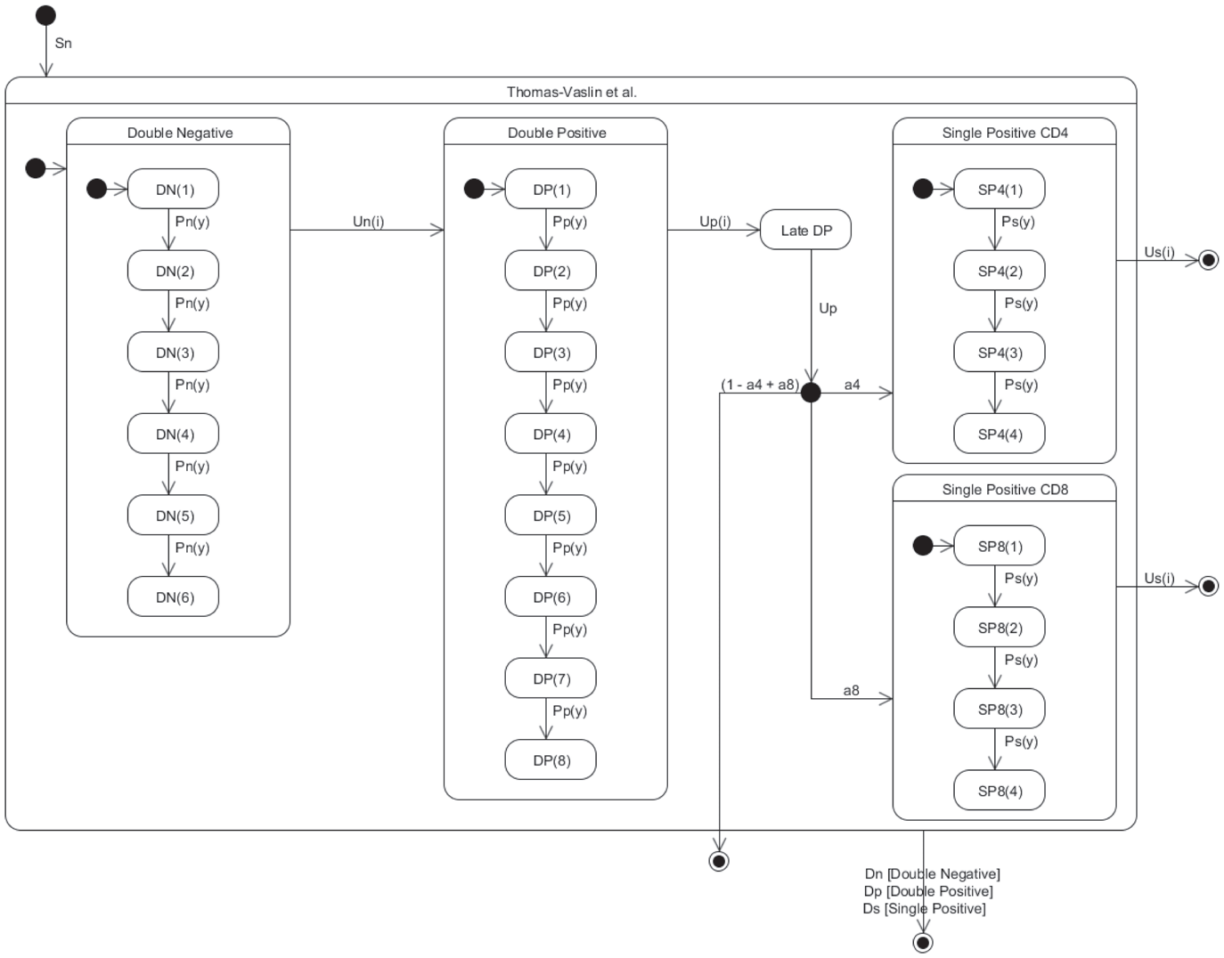
State-transition diagram of the Thomas-Vaslin et al. model [**15**] of thymocyte differentiation. This diagram attempts to essentially capture the same biological information as the one contained in figure 1b.



The Thomas-Vaslin et al. model [15] is a compartmentalized ordinary differential equation model. Figure 11 represents the state-transition description of the model, which largely reflects the conceptual “conveyor belt” models of T-cell differentiation classically admitted and schematically represented by immunologists [16]–[18]. Indeed the mental analogy between state transition and conveyor belt is obvious, reinforcing our conviction that immunologists (and biologists in general) should quite easily adopt the graphical language of state-transition diagrams. In essence, each stage of the conveyor belt represents a particular lineage and differentiation stage of T-cells, with flows into and from a particular stage according to the general equation:

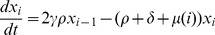
(34)


The interested reader is referred to the original paper since no attempt is done here to modify in any way the original model. 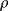, 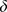 and 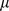 represent proliferation, death and differentiation, respectively, and 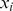 represents the 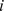
’th stage. The parameter 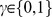 represents the possibility to deplete specifically dividing T cells, by the presence or absence of a pharmaco-genetic treatment, as explained in the original paper. Two daughters of a proliferating cell migrate into the next generational compartment, except during treatment (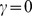) when dividing cells die by apoptosis and are lost from the model. The parameter 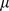 is an increasing function of generation, making cells more likely to differentiate between phenotypic compartments as they progress through their lineage. The model largely consists of constant progenitor influx (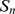); differentiation between thymocyte developmental phenotypes double negative (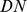) and double positive cells (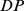) (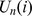 and 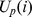); egression of single positive (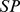 stage), either CD4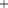 or CD8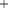 cells, to the periphery (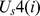, 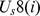); proliferation (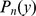, 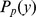, and 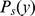); positive and negative selection (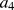, 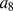) and natural cell death (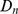, 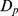, and 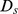). There is no parallelism in this model, reflecting the original formulation as a system of ordinary differential equations. However hierarchy and compound states are present clearly reducing diagram clutter. For instance, the transition 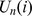 from *Double Negative* implicitly leaves all sub-stages of proliferation, *DN(1), DN(2)* and so on, that make up the *Double Negative* stage. The value of these differentiation transition rates depends on the successive stages of proliferation.

All 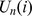 transitions enter the *Double Positive* stage in *DP(1)*, as indicated by the stub transition in solid black. The same convention applies to other transitions between compound states. Note that in all stages a cell can die naturally, but this need only be depicted once as a transition exiting the main chart, implicating all stages.

Intuitively, each variable in the system maps onto a stage in Fig. 11; each term in the equations maps onto a transition. Although the original model is composed of 30 quite similar differential equations, this whole mathematics and the code that captures it can easily be deduced and regenerated from the Fig. 11.

### State-transition Description of an ABM Model of Thymocyte Life Cycle in the Thymus

**Figure 12:**
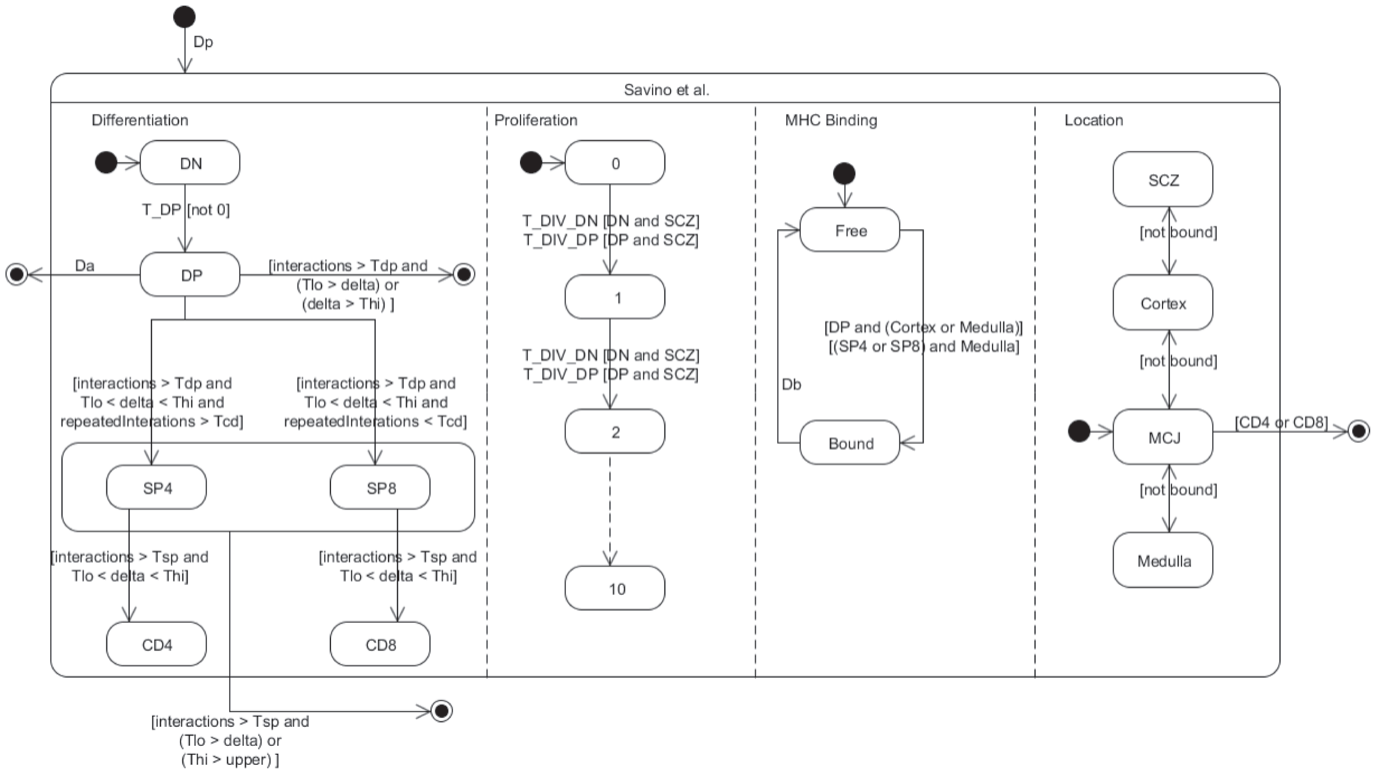
Statechart description of the Souza et al. model [**19**] depicting differentiation, proliferation, TCR-MHC binding and movement between the different anatomical compartments of the thymus. Although many transitions occur with parameterized rates, those related to differentiation are rule-based – making use of transition guards that refer to individual attributes. This model has no real hierarchical structure other than to conveniently specify a shared transition for SP4/SP8 cells.

Let’s now turn to the ABM Souza et al. model [19] which was originally proposed and simulated as an ABM cellular automata (CA). As a whole, the transition rules of any CA map naturally and elegantly onto a parallel state-transition diagram. Again, the interested reader is referred to the original paper for a detailed understanding of the simulation. Although available for download, the Fortran source code is far from easy to understand and the provision of the state-transition diagram, as done in this paper, should considerably improve the situation allowing the researchers to progress further with the existing simulator. The parallel state-transition diagram in Fig. 12 represents the different simultaneous transitions taking place in the model and coded as various CA rules: cells in the model differentiate and proliferate, they may be bound to thymic epithelial cells, they move and may be located in one of several anatomical compartments of the thymus. A complete description of the model includes additional implementation details that are abstractions of the mechanisms behind cell decisions to differentiate and apoptose. Many parameters characterizing the transition rules of the CA and which should be available for testing by the experimentalist appear in the state-transition diagram. Among others, the modeling abstractions employed in [19] and indicated in the state-transition diagram of Fig. 12 are.

- TCR-MHCp binding: a real-valued comparison of the difference between a T-cell receptor and an epithelial cell’s MHC-peptide complex, both abstracted as uniformly random numbers. Small differences between both numbers correspond to high affinity and vice-versa. At each interaction, a T-cell sums these differences and it is the value of this sum, specifically where it lies in relation to upper and lower threshold parameters, 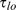 and 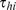, that determines whether the T-cell is positively or negatively selected. Double-positive and single-positive phenotypes have their own threshold parameters. Binding lasts for 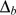 time.
- Number of interactions: an integer counter of how many interactions a T-cell has had with epithelial cells. Double- and single-positive cells cannot differentiate until they cross a threshold number of interactions, represented by the parameters 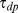 and 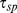, respectively. If, after 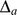 time a double-positive cell has not reached this threshold it apoptoses, i.e. death by neglect.
- Number of repeated interactions: an integer counter of how many times an individual T-cell has interacted with *the same* epithelial cell. This is Souza et al’s abstraction of the “signal-duration” hypothesis where long duration TCR-MHC interactions promote the CD4 phenotype and short duration promote the CD8 phenotype. Again, a threshold parameter 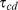 simulates the phenotype decision.

Although originally coded as an ABM CA simulation, nothing prevent a PBM version of this model, where populations instead of single cells occupy each site of the CA. This alternative might be as well automatically generated out of the diagram.

## Discussion

This paper advocates that with some minor enrichment of the UML state-transition diagrams to better align with scientific investigation and reporting, this standardized graphical language can serve as an effective medium of communication and development for both theorists and experimentalists. Despite many controversies still existing about the role played by the UML diagrams with regard to the final code production, no competent developer would deny the positive impact these diagrams can provide at particular phases of development. Theoretical biologists and immunologists [10], [11] could gain a lot from the adoption of standardized professional programming practices, rendering their software more readable, scalable and usable. Such adoption could end the current frustrating situation of “write once run only once” and save so many efforts in programming the same biological mechanisms again and again.

We have only concerned ourselves with a formalisable subset of statecharts, avoiding features unnecessary for our needs and, arguably, for scientific models. Although we have presented the formal constructs in the context of very didactic hypothetical examples, they all testify of some difficulties actually met in modeling immunological data that are inherently parallel and hierarchical using traditional techniques. This work is still in progress and we have some clear ideas where future efforts may focus.

Our non-classical use of statecharts leaves us wanting in some respects. For example, the informal referencing of parameters, variables, their units and the functional form of transitions are not entirely satisfactory. Largely, these issues are not ODE-specific and appear to be solvable with “syntactic sugar” rather than reinventing the statechart formalism under a different moniker.

Our focus has been on managing descriptive complexity, but computational complexity is an ever-present issue too. It is an interesting open question as to when highly compartmentalised ODE models (such as [15]) would become less efficient than a pure ABM effort (such as [19]). Certainly, although we can insulate the modeler and the experimentalist from the combinatorial growth of states, we cannot yet insulate the machine. For scientific simulation, this is a much less pressing issue than depicted in the statecharts literature, where systems are often embedded with realtime constraints, but exponential growth is still a concern in the long term. One interesting possibility is that the techniques used by statechart researchers (e.g. see [20]) may be adapted as hybridised numerical integration algorithms.

Statecharts can represent high-level semantics suitable for pedagogical descriptions as well as low-level quantitative information suitable for individual-based ABM and population-based ODE. Once adopted, they might contribute to ease the comprehension of ABM or Cellular Automata models proposed by more and more theoretical immunologists these days and to fill the gap still existing between these often opposed more top-down and more bottom-up simulation approaches [10]–[12].

A unified formalism would better allow these different levels of description to be directly compared, highlighting how each modelling effort realises the phenomenological description and the quantitative and structural assumptions it makes in doing so. This would require some novel enhancements to the statechart language, e.g. abstracting interactions that may be based on mass-action assumptions or localized cellular-automata neighborhoods, depending on the simulation method. This is largely a problem of transition implementation, although there are several subtle issues that would need to be resolved.

The ability to model sub-system parallelism and multi-level hierarchy is a powerful feature of UML Harel state-transition diagrams – a feature that has been somewhat lost in the literature under computer science and software engineering nomenclature. We have shown how a formalized subset of these diagrams can be applied to scaling mathematical ODE descriptions of systemic phenomena. In addition to finessing the associated increase in underlying model complexity for the modeler, this approach provides a graphical communication medium that, in our experience, is readily accepted by non-technical collaborators and can be productively discussed, questioned and reformulated without excessive concern for underlying technical details.

We believe that these state-transitions diagram complement existing methods in Systems Biology, allowing a systems approach to be taken at a coarser granularity than biochemical reactions, from various level of granularity from molecules to organisms [21]. We expect that such computer refactorized model not only will offer interoperability between models and improvement of them for enlarged integration of multiscale biological systems but also will allow to execute simulations to test their variability, fluctuations, robustness, emergence/immergence. Such progress is necessary in many domains, such as immunology, where the primary focus is on intra- *and* inter-cellular interactions. Of course, this argument generalizes to the scientific study of any complex system where “agents” are compound, stateful and inter-dependent, like ecological or social systems.
